# Impairment of neutrophilic glucocorticoid receptor function in patients treated with steroids for septic shock

**DOI:** 10.1186/s40635-015-0059-9

**Published:** 2015-07-28

**Authors:** Maria Bergquist, Catharina Lindholm, Morten Strinnholm, Göran Hedenstierna, Christian Rylander

**Affiliations:** Department of Medical Sciences, Clinical Physiology, Uppsala University, S-75185 Uppsala, Sweden; Department of Rheumatology and Inflammation Research, Sahlgrenska Academy, University of Gothenburg, Gothenburg, Sweden; Department of Anesthesia and Intensive Care, Kungälv Hospital, Kungälv, Sweden; Department of Anesthesia and Intensive Care, Sahlgrenska University Hospital, Gothenburg, Sweden

**Keywords:** Septic shock, Glucocorticoid receptor, Dexamethasone, Hydrocortisone, Cortisol, Human

## Abstract

**Background:**

Glucocorticoid (GC) treatment has variable effect in sepsis. This may be explained by decreased expression or function of the glucocorticoid receptor (GR). The aim of this study was to determine GR expression and binding capacity in patients during and after sepsis.

**Methods:**

In this prospective, non-interventional clinical study, peripheral blood and clinical data were collected from 20 adult patients at five timepoints during sepsis and 5–13 months after recovery. GR expression and binding capacity were assessed by flow cytometry.

**Results:**

GR expression was higher in T lymphocytes from patients with septic shock compared to healthy subjects (*p* = 0.01). While there was no difference in GR expression between GC-treated and non-treated patients, GR binding capacity was lower in GC-treated patients at admission compared to healthy subjects (*p* ≤ 0.03). After the acute inflammation inflammatory phase, GR binding capacity was still lower in neutrophils of GC-treated patients, compared to healthy subjects (*p* = 0.01). On admission, GR binding capacity in T lymphocytes and neutrophils was inversely correlated with noradrenaline dose and lactate (*p* ≤ 0.03).

**Conclusions:**

Our data suggest that GR expression is increased in T lymphocytes during septic shock regardless of GC treatment, while GR binding capacity is decreased in neutrophils in GC-treated patients. As neutrophils are the predominant circulating leucocyte in septic shock, their decreased GR binding capacity may impede the response to exogenous or endogenous glucocorticoids.

## Background

Steroid treatment for septic shock remains controversial due to contradictory results from clinical trials [1]. The main effects of endogenous and exogenous glucocorticoids (GC) are exerted through their binding to the intracellular glucocorticoid receptor (GR) present in all cells [2]. During early human sepsis, cortisol concentrations gradually rise with higher levels being inversely related to the chance of survival [3]. It is generally inferred that increased circulating cortisol during critical illness is a consequence of increased HPA axis activity and increased levels of ACTH, although ACTH levels have been found at similar levels or even below those of healthy controls [4–6]. These findings suggest that rather than an increased production of cortisol, elevated cortisol levels are a consequence of a dysfunctional cortisol clearance from circulation which results in the supranormal cortisol levels found in the critically ill patients. Indeed, the expression and activity of A-ring reductases (the principal route of cortisol breakdown in humans) and 11β-HSD type 2 (converting cortisol to cortisone, inert to cells) were found reduced in the liver but not in the adipose tissue [6]. This raises the question whether the glucocorticoid receptor also has a decreased expression or function during critical illness. The importance of GR was recently illustrated by increased mortality, hemodynamic instability, and pro-inflammatory cytokine production found in mice, specifically lacking endothelial GR, subjected to endotoxic shock [7]. Our group recently showed that GR binding capacity was decreased in neutrophils during endotoxic shock in mice [8]. Previous quantitative studies of GR in critical illness investigated mRNA expression levels [9, 10], which precludes conclusions about the protein expression and subsequent receptor function. To the best of our knowledge, the effect of human sepsis on GR protein expression and GR binding capacity has not been investigated previously. The aim of this explorative, observational study was thus to describe GR expression and binding capacity in circulating T lymphocytes and neutrophils, sampled from peripheral blood of adults during septic shock and after recovery.

## Methods

### Patients and methods

This multicenter study was approved by the Ethical Committee for Human Research in Uppsala, Sweden.

### Patients and healthy subjects

Twenty patients were recruited between February 2012 and May 2013 in the general intensive care units (ICU) of the Sahlgrenska University Hospital and the Kungälv Hospital. During this period, 146 patients with septic shock were treated in the two ICUs, where hydrocortisone is used according to the Surviving Sepsis Campaign guidelines [11] at the discretion of the clinician. The selection of patients was influenced in a non-systematic way over time by the availability of laboratory resources. Inclusion criteria were (i) age over 18, (ii) sepsis according to the American-European consensus criteria [12], and (iii) treatment with at least one vasopressor. Exclusion criteria were (i) known infection with human immunodeficiency virus or hepatitis B or C, (ii) chronic treatment with GCs, (iii) participation in any investigational drug study within 4 weeks preceding the study period, and (iv) survival expected to be shorter than 3 days. Informed consent was initially obtained via next of kin and later directly from survivors. Consenting healthy subjects were recruited among non-smoking laboratory and hospital staff without any chronic or acute illness and with no medication.

Blood and clinical data were collected three times during septic shock, at T0 within 24 h of ICU admission, T1 24 h after T0, and T2 48–120 h after T0 depending on logistics. Then, the blood was sampled when the acute inflammatory phase was considered to be over, and the patient was stable without support to vital functions at T3, 4–19 days after ICU admission. Finally, the blood was sampled after recovery at T4, 5–13 months after the patient had been discharged from the ICU. Survival was defined as alive 180 days after ICU admission. GR data from one patient at T0–T2 were lost due to technical reasons; one patient was lost to sampling at T3 and one to follow up at T4. One patient was excluded from sampling at T4 due to chronic GC treatment at that time. Clinical variables relevant for the degree of septic shock and organ dysfunction (blood pressure, heart rate, lactate, ScvO_2_, temperature, a-pH, base excess, serum creatinine, serum bilirubin, Glasgow Coma Scale (GCS) score, Simplified Acute Physiology Score (SAPS) III score, type and dose of vasoactive/inotropic agent) were registered for all timepoints where applicable. Blood gas values and clinical laboratory data (CRP, leucocyte and platelet counts, and microbiological cultures) were obtained from laboratory records. For plasma and serum, blood samples were centrifuged at 1500*g* for 15 min and plasma was stored in −70 °C until analysis.

### Cell preparation

Whole blood aliquots were centrifuged with phosphate-buffered saline (PBS) at 1500*g* and 4 °C for 10 min and subjected to erythrocyte lysis using BD FACS Lysing Solution (BD Biosciences, San José, CA, USA) for 15 min at room temperature. Leucocytes were then washed again twice with PBS and kept on ice until the total number of leucocytes was determined using an automated cell counter (Sysmex KX-21N, Kobe, Japan). For flow cytometry, a total of 5 × 10^5^ cells were added to each well on a polypropylene conical 96-well plate (Thermo Fisher Scientific, Rochester, NY, USA) and stained with fluorochrome-conjugated antibodies binding to cell surface markers after Fc-blockage (Beriglobin®, CSL Behring, Denmark), anti-CD4 v450 (RPA-T4, BD Horizon™, BD Biosciences), anti-CD19 PerCP (4G7), anti-CD3 PerCP (SK7, BD Biosciences), anti-CD56 PE (B159), anti-CD16 Alexa Fluor® 647 (3G8), anti-CD8 APC (RPA-T8), and anti-CD14 PE (M5E2 BD Pharmingen™). Surface staining was performed at +4 °C in the dark for 20 min, followed by washing twice in FACS buffer (PBS supplemented with 100 mM EDTA, 1 % fetal calf serum, and 0.1 % NaN3).

Cells were fixed and permeabilized for intracellular staining using a Fixation/Permeabilization kit (eBioscience, San Diego, CA, USA) at +4 °C for 30 min. After washing twice in permeabilization buffer, Fc-blockage was performed in permeabilization buffer at +4 °C in the dark for 10 min. GR was labeled using anti-GR mAb (5E4, AbD Serotec, Dusseldorf, Germany) in permeabilization buffer at +4 °C in the dark for 30 min, followed by two washes with permeabilization buffer. Fluorescein isothiocyanate (FITC)-labeled mouse IgG1 isotype control (G18-145, BD Pharmingen™) was used as a negative control. For analysis, the cell pellets were resuspended in FACS buffer.

A separate set of samples for analysis of GR binding capacity was surface stained as described above but not permeabilized. Instead, cell pellets were resuspended in 10 μL FACS buffer containing 20 nM FITC-conjugated dexamethasone (FITC-Dex) (Molecular Probes, Invitrogen, Carlsbad, CA, USA) [13]. Negative control samples were resuspended in 10 μL FACS buffer containing 20 nM dexamethasone (Merck, Darmstadt, Germany). After incubation at +37 °C in the dark for 30 min, cells were washed twice in FACS buffer and fixed with 4 % paraformaldehyde at room temperature for 15 min, then resuspended in FACS buffer and analyzed by flow cytometry. In an initial competitive experiment to exclude non-specific binding by FITC-Dex, cells were incubated with non-conjugated dexamethasone first, followed by a second incubation with FITC-Dex, which revealed a non-specific binding of less than 7 % (data not shown).

### GR analysis

GR expression and binding capacity in different leucocyte subsets were assessed using a FACS CantoII (BD Biosciences) equipped with Diva software (BD Biosciences). For each sample, at least 10,000 events were collected in the lymphocyte gate. Fluorescence was compensated using BD Anti-mouse CompBeads (BD Biosciences). For control of day-to-day variations of the assay, leucocytes from one healthy blood donor were included in each analysis. This internal standard was prepared sterile at one time as follows. Peripheral blood mononuclear cells (PBMCs) were separated using Lymphoprep (Fresenius Kabi, Oslo, Norway), washed in ice cold sterile PBS, and frozen in heat inactivated fetal calf serum with 15 % DMSO at a rate of −1 °C/min using a Nalgene® Mr. Frosty™ Freezing Container (Thermo Scientific, Waltham, MA, USA). For each analysis, a vial of the internal standard was defrosted on ice and washed in FACS buffer followed by surface and intracellular staining together with each patient sample. Data was further processed using FlowJo software (Tree Star, Inc., Ashland, VA, USA).

### Cytokine analysis

Cytokines, granulocyte colony-stimulating factor (G-CSF), intracellular adhesion molecule 1 (ICAM-1), interferon alpha (IFN-α), interferon gamma (IFN-γ), interleukin 1 alpha (IL-1α), interleukins (IL-1β, IL-4, IL-6, IL-8, IL-10, IL-12p70, IL-13, IL-17A), interferon gamma-induced protein 10 (IP-10), latency-associated peptide (LAP), monocyte chemotactic protein 1 (MCP-1), macrophage inflammatory protein 1 alpha and beta (MIP-1α, MIP-1β), tumor necrosis factor (TNF), and E-selectin, were analyzed in plasma using the Human Inflammation 20-plex RTU FlowCytomix Kit (eBioscience) according to manufacturer’s recommendations. In short, 25 μL of plasma was incubated with the bead mixture and biotin-conjugate at room temperature for 2 h, shaking at 500 rpm. After wash, the samples were incubated with 50 μL Streptavidin-PE solution at room temperature for 1 h, shaking at 500 rpm. After final washes, the samples were analyzed in 280 μL assay buffer using a FACS CantoII (BD Biosciences).

### Cortisol analysis

For quantification of cortisol, plasma was analyzed using a commercially available EIA kit (DetectX® Cortisol, Arbor Assays, MI, USA), according to manufacturer’s instructions. Optical density was read at 450 nm using a Spectra Max 340PC (Molecular Devices, Sunnyvale, CA, USA) running the SoftMax Pro 5.2 software.

### Statistical analysis

Results were graphically presented with individual plots as well as group geometric means. Grouping of patients into GC-treated and GC-non-treated was based on GC treatment having been instituted or not at the time when samples were taken. Differences between groups were tested with the two-tailed Mann-Whitney test (GaphPad Prism 6.0 for Windows, GraphPad Software Inc, La Jolla, CA, USA). Associations between clinical parameters and GR expression or binding capacity at T0 in T lymphocytes and neutrophils were evaluated using the Spearman correlation test. The analyses were done using the R environment for statistical computing (version 3.1) [14] and the coin add-on package (version 1.1) [15]. *p* values ≤ 0.05 were considered statistically significant. Given the explorative nature of the study, multiple comparisons were not corrected for.

## Results

### Patient and healthy subject characteristics

Characteristics of the patients and the healthy subjects are listed in Table 1. Clinical parameters related to severity of shock are listed in Table 2. Fourteen of the 20 patients were treated with GC at some point, and six patients did not receive GC at any time. Two patients were GC-non-treated until T2 and T3, respectively. Of the clinical parameters, the plasma level of lactate and the dosage of noradrenaline were higher in the GC-treated than in the GC-non-treated patients at T0 (*p* = 0.004 and 0.01, respectively). Other shock-related parameters, however, did not differ between these groups. Out of 14 GC-treated and 6 GC-non-treated patients, 5 and 3 were dead after 180 days, respectively.

**Table 1:** Demographics of patients and healthy subjects

Parameter	GC (*n* = 14)	No GC (*n* = 6)	Healthy subjects (*n* = 16)
Men/women	7/7^a^	3/3	3/12^b^
Age, mean (SD) years	62.2 (12.3)	62.3 (19.1)	45.4 (11.1)
Source of sepsis^c^			
Lungs	9	1	
Abdomen	2	2	
Wound	1	2	
Urinary tract	2	1	
Unknown	0	1	
SAPS III, mean (SD)	67.6 (17.2)	65.9 (15.1)	
Alive/all at			
T0	12/12	8/8	
T1	12/12	8/8	
T2	13/13	7/7	
T3	12/14	6/6	
T4	9/14	3/6	

*GC* patients treated with glucocorticoids, *No GC* patients not treated with glucocorticoids at any time

^a^Two patients were GC-non-treated until T2 and T3, respectively

^b^Gender and age were not registered from one healthy subject

^c^One patient had sepsis from two suspected sources

**Table 2:** Clinical data related to shock

Parameter	GC			No GC		
	T0	T1	T2	T0	T1	T2
Noradrenaline dose [μg/kg/min]	0.45 (0.12, 0.87)^a^	0.55 (0, 1.46)^a^	0.18 (0, 1.1)	0.15 (0.09, 0.25)	0.10 (0, 0.32)	0.05 (0, 0.23)
Mean blood pressure [mmHg]	71 (60, 88)	68 (55, 90)	83 (66, 112)	69 (34, 90)	75 (62, 90)	86 (70, 100)
Heart rate [bpm]	105 (75, 138)	106 (35, 125)	92 (69, 120)	92 (66, 146)	96 (55, 140)	78 (65, 96)
ScvO_2_ [%]	74 (57, 89)	73 (48, 89)	76 (47, 91)	79 (69, 85)	75 (70, 87)	72 (63, 76)
Temperature [°C]	37.3 (35.4, 38.3)	37.6 (36.3, 38.8)	37.0 (35.7, 38.7)	37.3 (33.0, 38.9)	36.5 (33.3, 38.4)	36.6 (34.9, 37.9)
a-pH	7.29 (7.18, 7.43)	7.31 (7.13, 7.49)	7.34 (7.22, 7.45)	7.35 (7.26, 7.42)	7.35 (7.26, 7.47)	7.37 (7.30, 7.41)
Base excess	−5.8 (−10, 1.3)	−3.9 (−15, 4.3)	0.4 (−5.4, 8.0)	−3.1 (−6.8, 0.8)	−1.6 (−10, 6.6)	0.2 (−2.8, 3.4)
Lactate	5.5 (1.3, 14)^a^	3.3 (1.1, 8.6)	1.8 (0.8, 3.8)	2.3 (1.3, 3.3)	1.9 (1.0, 3.2)	1.3 (0.6, 2.2)
LPK	8.3 (1.3, 30)	11 (1.3, 23)	12 (1.5, 22)	14 (1.3, 28)	11 (3.3, 24)	9.4 (3.9, 18)
TPK	192 (52, 366)	125 (34, 277)	67 (9.4, 148)	192 (29, 473)	157 (28, 343)	133 (30, 239)
CRP	167 (49, 290)	215 (52, 410)	154 (46, 330)	213 (140, 336)	247 (92, 328)	151 (46, 315)

Numbers are given as median (range)

^a^Difference between patients treated with glucocorticoids (GC) and patients not treated with glucocorticoids (No GC), *p* < 0.05

### Glucocorticoid receptor expression is elevated in T lymphocytes

**Fig. 1:**
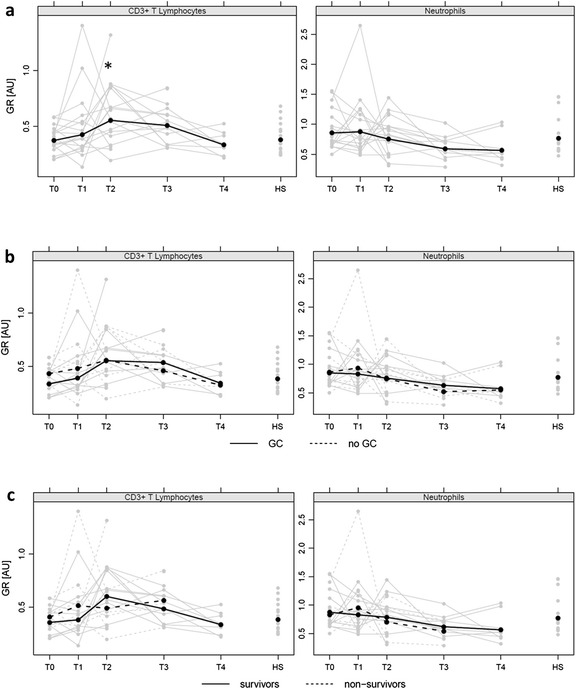
Glucocorticoid receptor (*GR*) expression in T lymphocytes and neutrophils from peripheral blood sampled during the initial phase of sepsis (*T0–T2*), after the acute inflammatory phase (*T3*), after recovery (*T4*), and from healthy subjects (*HS*). GR expression was determined by flow cytometry as mean fluorescence intensity (MFI) and normalized to an internal standard. **a** *Plots* represent group means (*black*) and individual patients (*gray*). **b** Patients divided into groups as glucocorticoid (*GC*)-treated and non-treated. Two patients were GC-non-treated until T2 and T3, respectively. **c** Patients divided into groups as survivors and non-survivors at 180 days. Difference between patients and healthy subjects (*HS*) was tested using the two-tailed Mann-Whitney test. *p < 0.05

The GR expression in T lymphocytes and neutrophils showed a high intra-interindividual variation among patients as illustrated in Fig. 1a. Compared to healthy subjects, GR expression was higher in T lymphocytes (*p* = 0.01) but not in neutrophils of patients during the shock phase (T2). After recovery (T4), GR expression in T lymphocytes had retroceded to the level seen in healthy subjects. There was no difference in GR expression between GC-treated and GC-non-treated patients or between survivors and non-survivors at the different timepoints (Fig. 1b, c). However, the relative increase during septic shock (T0–T2) was higher in CD4+ T lymphocytes of survivors compared to non-survivors (*p* = 0.03). At T0, GR expression in T lymphocytes did not correlate with any of the clinical parameters, but in neutrophils, it was inversely correlated with a-pH (*p* = 0.03) and base excess (*p* = 0.02).

### Glucocorticoid receptor binding capacity is reduced in neutrophils

**Fig. 2:**
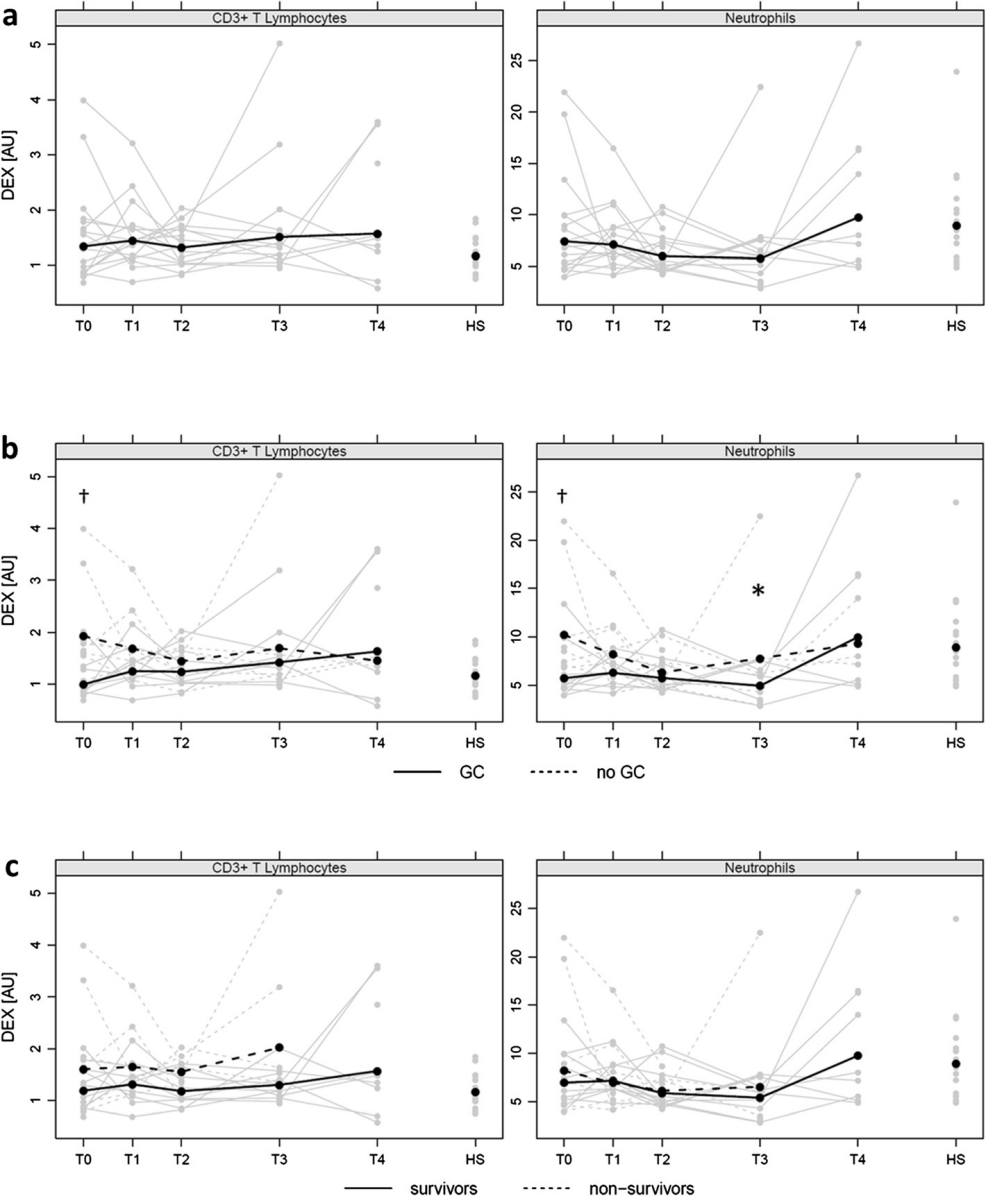
Glucocorticoid receptor (*GR*) binding capacity in T lymphocytes and neutrophils from peripheral blood sampled during the initial phase of sepsis (*T0–T2*), after the acute inflammatory phase (*T3*), after recovery (*T4*), and from healthy subjects (HS). GR binding capacity (*DEX*) was determined by flow cytometry as mean fluorescence intensity (MFI) of fluorescence-labeled dexamethasone and normalized to an internal standard. **a** *Plots* represent group means (*black*) and individual patients (*gray*). **b** Patients divided into groups as glucocorticoid (*GC*)-treated and non-treated. Two patients were GC-non-treated until T2 and T3, respectively. **c**. Patients divided into groups as survivors and non-survivors at 180 days. Difference between patients and healthy subjects (*HS*) was tested using the two-tailed Mann-Whitney test. *p < 0.05. Difference between GC-treated and non-treated patients was tested using the two-tailed Mann-Whitney test. ^†^ *p* < 0.01

**Fig. 3:**
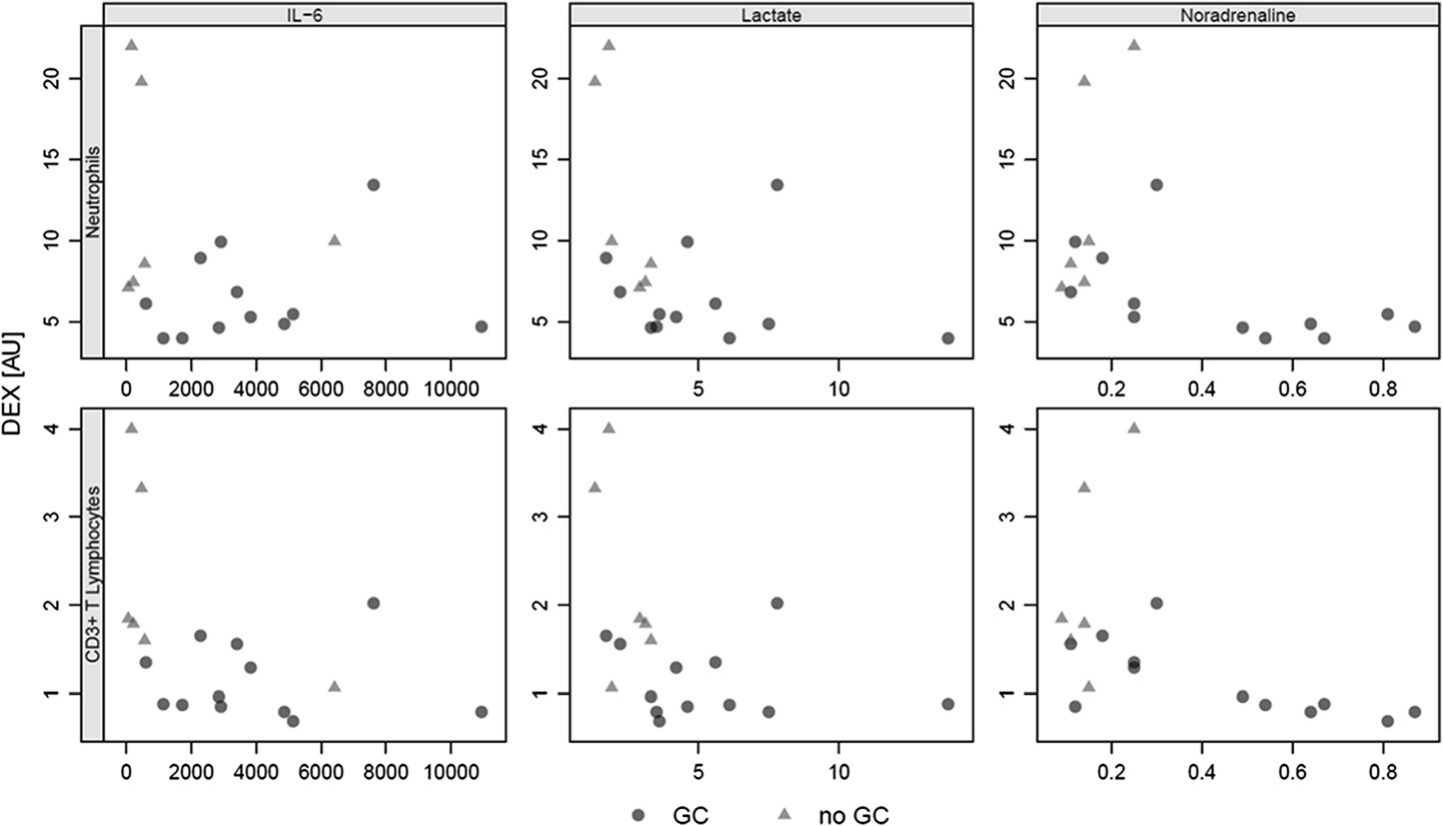
*Scatter plots* representing inverse correlations between plasma IL-6 (pg/mL), lactate and noradrenaline dose and GR binding capacity (DEX) in T lymphocytes (p ≤ 0.02) and noradrenaline dose and lactate in neutrophils (*p* ≤ 0.03) in all patients at admission (T0). Associations between clinical parameters and GR binding capacity were evaluated using the Spearman correlation test

The GR binding capacity in all patients is illustrated in Fig. 2a. During septic shock, it did not differ from the GR binding capacity found in healthy subjects. However, when the acute inflammation had ceased (T3), GR binding capacity was lower in neutrophils of GC-treated patients compared to healthy subjects (*p* = 0.01). At the first measurement after admission (T0), GR binding capacity was lower in T lymphocytes (*p* = 0.008) and neutrophils (*p* = 0.03) of GC-treated patients compared to GC-non-treated patients (Fig. 2b). Non-survivors and survivors differed (*p* = 0.04) in evolution of GR binding capacity in neutrophils as non-survivors showed a relative increase while survivors showed a relative decrease between septic shock (T0) and their stabilized condition (T3). At T0, GR binding capacity in T lymphocytes was inversely correlated with IL-6, noradrenaline dose, and lactate (*p* ≤ 0.02), and in neutrophils, it was inversely correlated with noradrenaline dose and lactate (*p* ≤ 0.03) (Fig. 3).

### T lymphocyte numbers remain low after recovery

The numbers of leucocytes on admission (T0) and after sepsis recovery (T4) are presented in Table 3. T lymphocytes were decreased, and neutrophils were increased after admission in septic shock (T0). After recovery (T4), patients treated with GC during sepsis still showed lower numbers of T and B lymphocytes compared to healthy subjects while neutrophil numbers were similar to the healthy subjects in both groups.

**Table 3:** Numbers of leucocytes per mL of peripheral blood

	Patients				Healthy subjects
	On admission (T0)		After recovery (T4)		
	No GC	GC	No GC	GC	
CD3+ T lymphocytes	2.3 (2.6)***	3.6 (4.9)***	11 (4.6)	9.4 (6.7)*	18 (7.9)
CD4+ T lymphocytes	1.1 (1.1)***	2.3 (4.3)***	5.1 (1.4)	4.3 (3.9)*	10 (4.4)
CD8+ T Lymphocytes	1.0 (1.1)***	1.3 (1.8)***	4.1 (2.7)	4.4 (5.6)	6.5 (3.2)
B lymphocytes	1.6 (2.7)	1.0 (1.7)	1.9 (2.0)	0.6 (0.7)*	2.0 (1.3)
NK cells	0.8 (1.4)	0.8 (0.7)	3.6 (2.3)	0.8 (0.8)	2.1 (2.0)
Monocytes	5.7 (3.5)	1.9 (1.8)***^a^	3.0 (2.1)	3.8 (2.5)	5.3 (2.5)
Neutrophils	145 (63)***	76 (66)^a^	28 (8.3)	41 (29)	51 (23)
Eosinophils	0.9 (1.2)	0.8 (2.2)*	1.8 (0.4)	2.0 (1.4)	2.9 (2.6)

Numbers are ×10^5^, given as mean (SD)

^a^Difference between patients treated with glucocorticoids (GC) and patients not treated with glucocorticoids at any time (No GC), *p* ≤ 0.05

*Difference between patients and healthy subjects (HS), *p* ≤ 0.05, ***p < 0.001

### Cortisol and cytokine response

**Fig. 4:**
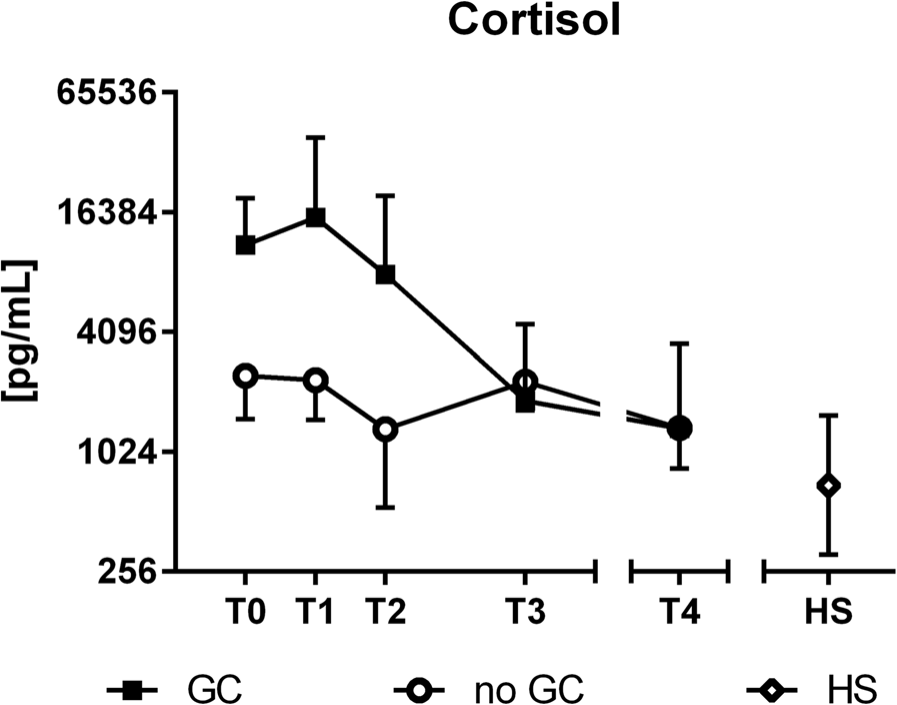
Plasma cortisol during the initial phase of sepsis (*T0–T2*), after the acute inflammatory phase (*T3*), after recovery (*T4*), and in healthy subjects (*HS*). Cortisol was analyzed by enzyme immunoassay (EIA). *Plots* represent geometric means with confidence intervals. *GC* glucocorticoid treatment, *HS* healthy subjects

**Fig. 5:**
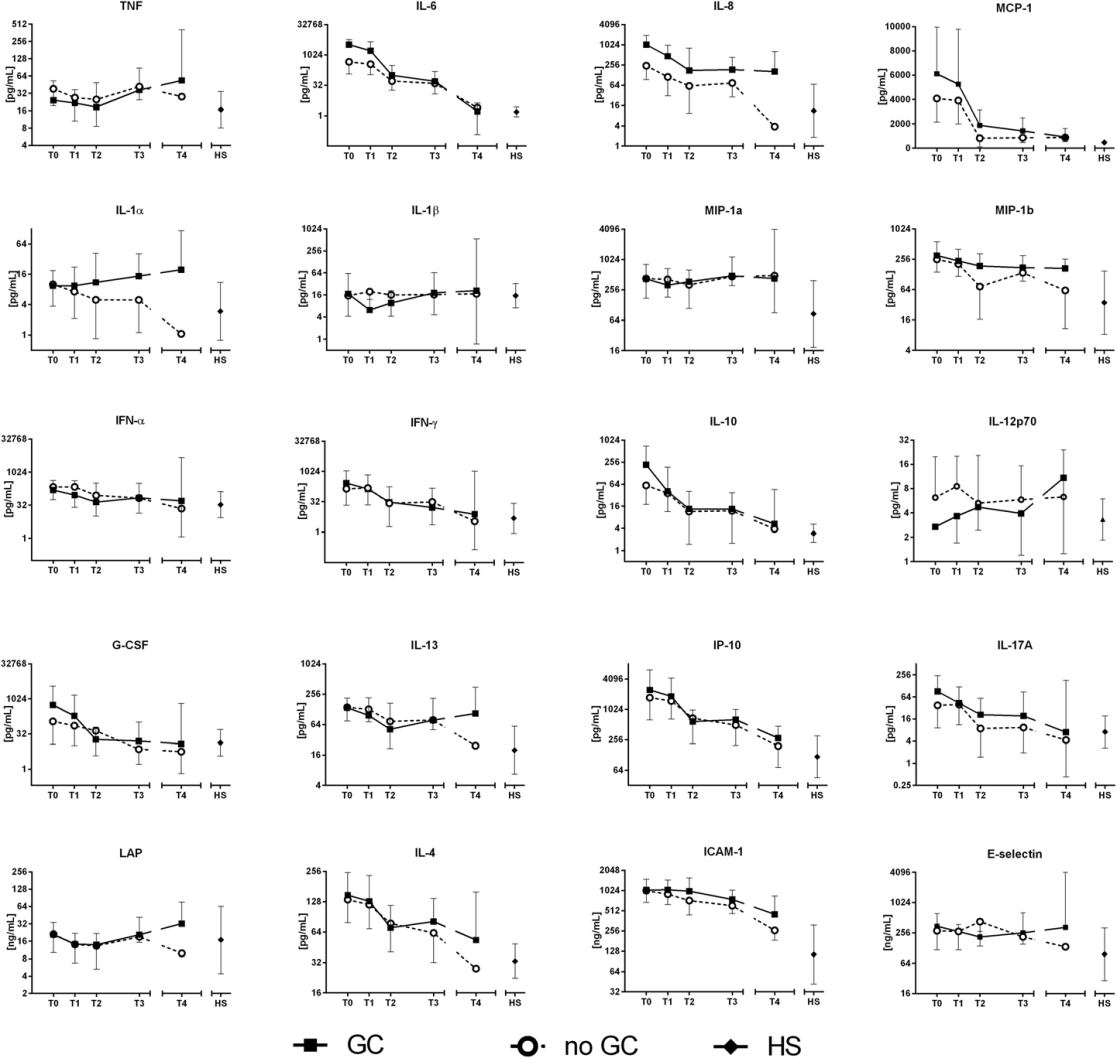
Plasma cytokines during the initial phase of sepsis (*T0–T2*), after the acute inflammatory phase (*T3*), after recovery (*T4*), and in healthy subjects (*HS*). Cytokines were analyzed by flow cytometry. *Plots* represent geometric means with confidence intervals. *GC* glucocorticoid treatment, *HS* healthy subjects

Shortly after ICU admission (T0), there were high levels of cortisol present in GC-treated patients as compared to healthy subjects (*p* = 0.01), reflecting both endogenous cortisol and hydrocortisone (Fig. 4). Most of the 20 cytokines analyzed displayed the highest concentration at T0, followed by a decline to levels similar to those of healthy subjects (Fig. 5). There were no significant differences between GC-treated and GC-non-treated patients although IL-6, IL-8, MCP-1, G-CSF, IL-17A, and IL-10 appeared higher in GC-treated patients, whereas TNF and IL-12p70 appeared higher in GC-non-treated patients at T0.

## Discussion

The main findings of this study are that GR expression is upregulated in T lymphocytes during septic shock regardless of steroid treatment and that GR binding capacity is decreased in neutrophils after the inflammatory phase in patients who have been treated with steroids.

An upregulation of GR expression seems plausible as part of an adaptive response to dampen an aggressive inflammation in the presence of invading pathogens. Indeed, we previously have found increased GR protein expression in circulating leucocytes and splenocytes in experimental endotoxic shock in mice [8]. However, in earlier studies of GR mRNA, sepsis and septic shock were associated with decreased GR expression. According to Ledderose et al., using T lymphocytes from adult sepsis patients found a lower, however, not statistically significant, expression of GR-α than in healthy subjects [9]. van den Akker et al. studied neutrophils sampled from children within 24 h after admitted to a pediatric ICU for sepsis and septic shock and found that GR-α expression was slightly depressed but not statistically different from healthy subjects. However, GR expression increased with recovery to significantly higher levels than were found on admission [10]. These opposite findings may represent inconsistency between the GR protein and mRNA concentration as measures of GR expression. In an abnormal inflammatory situation such as sepsis, it is plausible that the speed of GR expression at the gene and protein levels can be restricted by a specific limiting factor. In eukaryotes, while mRNA can be produced in two copies per hour, dozens of the corresponding protein can be translated in the same time [16, 17]. Transcription is estimated to be slower than translation because of several time-consuming steps, such as transcription initiation, intron excision, and posttranscriptional RNA processing, which are not required for protein translation. In addition, proteins have longer half-life than mRNAs (46 h versus 2.6–7 h, respectively) [17].

Furthermore, we observed that survivors had a relatively higher increase of GR expression in T lymphocytes during septic shock than non-survivors. This clinical finding is supported by experimental results from Goodwin et al. who found that GR knockdown in endothelial cells proved to increase mortality and hemodynamic instability in mice subjected to endotoxic shock [7]. The importance of GR expression for survival of septic shock has been further demonstrated by Kleiman et al. who showed that GR dimerization-deficient mice were highly susceptible to endotoxic shock, suggestively by their inability to downregulate the pro-inflammatory cytokine IL-1β [18]. With our method, we did not investigate GR dimerization in itself but a high GR expression, as found in survivors, is a prerequisite for sufficient dimerization and function of the receptor.

The other, and perhaps most important finding in this study, was that GR binding capacity was decreased in neutrophils of patients treated with steroids. These patients were administered 200 mg of hydrocortisone daily in line with current guidelines [11]. Their GR binding capacity was significantly lower than in healthy subjects at admission, but also, after septic shock was resolved. Impaired GR function has been described previously, in peripheral blood mononuclear cells in sepsis [19], lung tissue in experimental acute lung injury [20], and by our group in neutrophils in experimental endotoxic shock [8]. One possible interpretation would be that the GC-treated patients were in a deeper state of shock than the non-treated patients, causing decreased GR binding capacity. However, this explanation is unlikely as the patients from both groups were similar in most shock-related parameters and also because the difference between GC-treated and non-treated patients still remained at T3, when the shock was resolved. With the recent observation of substantial reduction in cortisol metabolism during critical illness [6], it is more likely that an excessively high concentration of endogenous cortisol and administered hydrocortisone caused decreased GR binding capacity. As the effects of signaling from the activated GR-GC complex during severe inflammation includes decreased capillary permeability, dampened cytokine release, and modulated leucocyte recruitment [21, 22], impaired GR binding capacity may have negative short-term consequences. Considering decreased binding capacity as well as the expanding number of neutrophils in sepsis, the inability of neutrophils to respond to endogenous or exogenous glucocorticoids may have severe ramifications for the host. In the presence of infection, while T lymphocytes display an increased GR expression, neutrophils may become resistant to glucocorticoid-induced apoptosis through decreased GR binding capacity. This is likely protecting the host from immunosuppression, which would exacerbate the present infection.

Our study also showed a severe retraction in the number of circulating T lymphocytes during sepsis. This is in agreement with previous studies [23] and may be an effect of the increased predisposition of T lymphocytes to undergo apoptosis in sepsis [24]. Neutrophils, on the other hand, were significantly increased in numbers during sepsis. Other studies have suggested that neutrophils are less sensitive to glucocorticoids than other leucocytes [25, 26]. Indeed, GR binding capacity in neutrophils was decreased by sepsis in our study. Taken together, these data suggest that T lymphocytes are the main glucocorticoid-responsive leucocytes, but may not be able to transmit an adequate effect of endogenous or exogenous glucocorticoids during sepsis due to their reduced number. Moreover, neutrophil activation is the predominant source of tissue damage and the multiple organ dysfunction syndrome (MODS) in critically ill patients [27, 28]. The acquired decreased binding capacity of neutrophils may be detrimental in sepsis and may even be aggravated by the increased circulating levels of glucocorticoids.

Interestingly, several months after recovery, survivors who received hydrocortisone treatment during sepsis in our study still had reduced numbers of CD3+ and CD4+ T lymphocytes and B lymphocytes well after recovery. GC-treated patients also showed higher concentrations of some cytokines at the time of recovery compared to any earlier timepoint and to healthy subjects. This is, to our knowledge, a novel observation. No patient displayed any symptom or sign of inflammation when blood samples were drawn after recovery, and it is therefore unlikely that they harbored any latent infection. If a decrease in T and B lymphocytes after sepsis recovery can be confirmed, this may explain why having survived sepsis is a strong predictor of developing subsequent infections [29]. Possible reasons for these findings remain speculative, and further studies are needed to confirm and delineate possible long-term cellular effects of glucocorticoid treatment during sepsis.

To the best of our knowledge, the expression and binding capacity has not previously been assessed on the protein level in septic shock. The method used in this study was partly developed by our group and is robust, offering information about both the GR expression at the protein level as well as its functional binding capacity ex vivo. It does not reveal information about the GR-α and GR-β isoforms. As the GR-α and GR-β isoforms originate from alternative splicing of the same gene, the only accurate way of specifically quantifying the GR-β isoform is using qPCR, which precludes information about both protein expression and function. For the present study, we chose to detect total GR expression and function using an antibody and fluorescence-labeled dexamethasone, respectively. The decreased GR binding capacity, that we observed using fluorescence-labeled dexamethasone, is unlikely to be an effect of competitive binding of hydrocortisone ex vivo. Naturally, occurring glucocorticoids (cortisol and hydrocortisone) dissociate within 15 min from GR, and any remaining unbound fraction would be washed away in the laboratory process. In addition, synthetic glucocorticoids have higher affinity for the receptor [30].

One limitation of the study is the non-consecutive inclusion (depending on available laboratory resources) of a relatively small study population, especially at the last timepoint (T4), and the demographic differences between patients and healthy subjects. The mean age of the healthy subjects was significantly lower, and immunological competence is known to decrease with age [31]. In addition, there were more women than men among the healthy subjects. However, when dividing the healthy subjects into two groups according to age or gender, we found no differences in GR expression or binding capacity. This does not preclude that age or gender had effects on the immune response in the patients, but they did not seem to affect our primary measured parameters in the healthy subjects. As this was an exploratory study, we did not make any multiplicity adjustments. Therefore, *p* values should be interpreted with caution due to the increased risk for false positives. To the best of our knowledge, the expression and binding capacity has not previously been shown in septic shock. We consider the observations in this exploratory hypothesis-generating study interesting enough to merit validation in a larger hypothesis driven study, testing the correlation between GR binding capacity and outcome.

## Conclusions

In summary, our data suggest that GR expression is increased in T lymphocytes during septic shock regardless of GC treatment, while GR binding capacity is decreased in neutrophils in GC-treated patients. As neutrophils are the predominant circulating leucocyte in septic shock, their decreased GR binding capacity may have severe consequences for the response to exogenous or endogenous glucocorticoids. Further studies are needed to investigate possible long-term immune traces of glucocorticoid treatment during sepsis.

## Abbreviations

DEX: dexamethasone
GC: glucocorticoid
GR: glucocorticoid receptor
